# Prevalence of allergen sensitization among asthmatic patients with serum total IgE >1000 IU/mL

**DOI:** 10.1002/clt2.70034

**Published:** 2025-02-11

**Authors:** Wanjun Wang, Lulu Wu, Jing Li, Qiurong Hu

**Affiliations:** ^1^ Department of Allergy and Clinical Immunology The First Affiliated Hospital of Guangzhou Medical University Guangzhou China; ^2^ Department of Respiration The First Affiliated Hospital of Guangzhou Medical University Guangzhou China

To the Editor,

Allergen sensitization occurs in most patients with asthma. Our previous article had demonstrated that house dust mite remained the most important allergen in Chinese individuals with asthma.^1^ A raised serum IgE against Aspergillus antigens usually occurred in bronchial asthma, especially a value ≥1000 IU/mL was recommended as the serum total IgE (tIgE) cut‐off to diagnose Allergic bronchopulmonary aspergillosis (ABPA).^2^ Therefore, we aim to describe the prevalence of sensitization to common allergens among asthmatic patients with serum tIgE >1000 IU/mL, the extent to which allergy accounts for these individuals is controversial.

We retrospectively analyzed the laboratory records of 1367 physician‐diagnosed asthma patients at the Department of Allergy and Clinical Immunology and Respiratory Medicine, the First Affiliated Hospital of Guangzhou Medical University, who had serum IgE test with a battery of common allergens performed (ImmunoCAP, ThermoFisher) during a 5‐year period from January, 2018 through October, 2024. All the patients analyzed had a blood tIgE level >1000 IU/mL. The allergens tested were house dust mite (*Dermatophagoides pteronyssimus*), grass pollen mix, food mix, cat, dog, *Alternaria alternata*, Aspergillus and *Penicillium notatum*. For patients who had multiple measurements performed during the study period, only the first time total serum IgE and specific IgE value were included for analysis. The criteria were excluded for patients with chronic obstructive pulmonary disease, helminth infection, rheumatic disease and tumors. This study was approved by the hospital ethics committee and the need for informed consent was waived.

Our study showed that all the patients were sensitized to at least two allergens and 734 (53.7%) individuals sensitized to more than 5 allergens. Allergic multimorbidities were very commonly found in nearly 98% of all patients’ allergies. Details about the baseline characteristics for patients are in Appendix S1. *Dermatophagoides pteronyssimus* (69.4%), *Aspergillus fumigatus* (29.7%), and cat (24.9%) were the three most common positive reactions in the tIgE >1000 individuals. Sensitization to inhalant allergens was significantly common (97.8%) as compared with food allergens (2.2%). The respective proportions for grass pollen, dog, *A. alternata* and *P. notatum* were 7.3%, 10.3%, 17.6% and 4.9% (Figure 1). In addition, a graded effect was observed with the serum tIgE level in these patients increasing with the number of positive allergens (*r* = 0.34, *p* = 0.047), and the *D. pteronyssimus* sIgE level (*r* = 0.52, *p* = 0.036). However, there was no statistically significant correlation between serum tIgE and Aspergillus sIgE level (Appendix S2).

**FIGURE 1:**
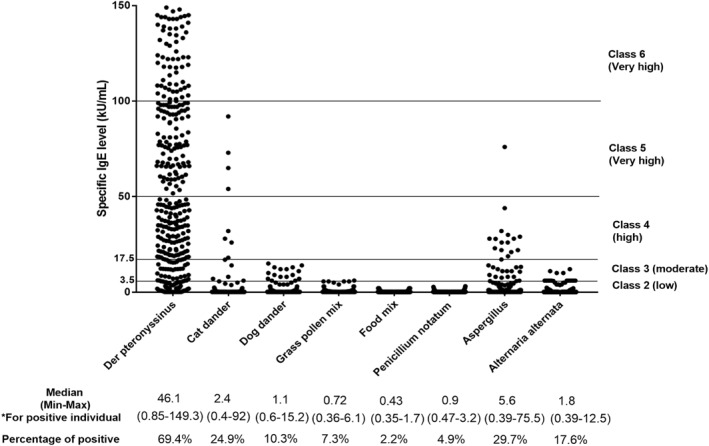
Levels of sIgE against the common allergens tested in a cohort of 1367 Chinese asthmatics. sIgE titers are expressed as kU/mL. The strength of IgE reactivity toward the specific allergens tested is categorized in classes from 0 to 6 according to ImmunoCAP standards as follows: class 0 (<0.35 kU/mL), class 1 (0.35–0.70 kU/mL), class 2 (0.70–3.50 kU/mL), class 3 (3.5–17.5 kU/mL), class 4 (17.5–50 kU/mL), class 5 (50–100 kU/mL), and class 6 (>100 kU/mL). The cutoff value was set at 0.35 kU/mL. The response was defined as positive if the sIgE level was ≥0.35 IU/mL. The species of the allergen source is listed below together with the rate of individuals positive for sIgE to each allergen tested. *The median sIgE titer including minimum (min) and maximum (max) is calculated for reactive individuals. sIgE, specific immunoglobulin E.

Our study focused on whether a fungal allergy must be present in asthma with elevated tIgE, and found that both house dust mites and cat dander were strong sensitizers. We observed that the *D. pteronyssimus* was still a major perennial allergen source and a significant cause of allergic asthma even in the high tIgE individuals. Some research found that asthmatics with atopic eczema and more allergic multimorbidities would preferentially develop strong Th2 responses against common allergens, such as mite proteases and Fel d1,^3^, ^4^ then the Th2 response promotes massive IgE release by plasma cells. Our hospital was a tertiary center of Southern China, which had enriched population of asthmatic patients from nationwide. Though ABPA was well recognized in refractory asthma, the prevalence of ABPA among asthmatics with tIgE >1000 IU/mL was 29.7% in our cohort. This finding was consistent with a similar study demonstrating that compared to non‐ABPA with a tIgE level >1000 IU/mL, the coexisting atopic diseases might influence the development of ABPA.^5^ Further large‐scale studies across different geographic regions are warranted to validate our findings. In conclusion, the current study suggests that the possibility of other allergen cosensitization should be clinically considered besides Aspergillus sensitization in these patients.

## AUTHOR CONTRIBUTIONS

**Wanjun Wang**: Writing—original draft; validation; data curation. **Lulu Wu**: Methodology; investigation; formal analysis. **Jing Li**: Funding acquisition; project administration; supervision. **Qiurong Hu**: Conceptualization; resources; writing—review and editing. Wanjun Wang and Lulu Wu shared first authorship. Jing Li and Qiurong Hu contributed equally as corresponding authors.

## CONFLICT OF INTEREST STATEMENT

The authors declare no conflicts of interest.

## Supporting information

Supporting Information S1

Supporting Information S2

## Data Availability

The data that support the findings of this study are available from the corresponding author upon reasonable request.
