# Supplementary material for: Prevalence of allergen sensitization among asthmatic patients with serum total IgE >1000 IU/mL
Source: Clin Transl Allergy. 2025 Feb 11;15(2):e70034. doi: 10.1002/clt2.70034 (PMC11813717; doi:10.1002/clt2.70034)
Supplement: Supplementary file 1 — Supporting Information S1 [file CLT2-15-e70034-s002.docx]

| **Items** |  |
| --- | --- |
| Gender M/F (cases) | 640/727 |
| Age distribution (y) | 31.8±22.3 |
| Children (≤14) | 52 (11.4±0.9) |
| Adults (>14) | 1315 (38.6±23.1) |
| Case distribution cases, n(%) |  |
| Asthma | 28 (2) |
| Asthma combined with* | 1339 (98) |
| Allergic rhinitis | 1298 (95.0) |
| Chronic rhinosinusitis | 672 (49.2) |
| Atopic dermatitis or urticaria | 174 (12.7) |
| Number of multimorbidity ≥ 3 | 632(46.2) |
| Clinical examination |  |
| FEV1 (%predicted) | 73.24±9.25 |
| FVC (%predicted) | 84.65±5.8 |
| FEV1/FVC | 81.5±7.5 |
| Total IgE (kU/L) | 4073.6±2786.9 |
| Eosinophil count (10^9^/L) | 0.83±0.35 |
| Asthma control test score （ACT） | 20.2±1.9 |
| Number of allergen sensitizations, n(%) |  |
| 1 | 0 (0) |
| 2 | 60 (4.4) |
| 3 | 254 (18.6) |
| 4 | 318 (23.3) |
| ≥5 | 734 (53.7) |

Appendix S1. Baseline information for 1367 asthmatic patients in the study.

Data are presented as mean±SE. FEV1, forced expiratory volume at 1s; FVC, forced vital capacity; IgE, immunoglobulin E.

*The figure of asthma combined with other allergic disorders had overlap.
