# Supplementary material for: Prevalence of allergen sensitization among asthmatic patients with serum total IgE >1000 IU/mL
Source: Clin Transl Allergy. 2025 Feb 11;15(2):e70034. doi: 10.1002/clt2.70034 (PMC11813717; doi:10.1002/clt2.70034)
Supplement: Supplementary file 2 — Supporting Information S2 [file CLT2-15-e70034-s001.docx]

A)

B)

Appendix S2. Correlation of tIgE levels with number of sensitized allergens, Dermatophagoides-sIgE levels, and Aspergillus-sIgE levels. The figures of number of sensitized allergens, Dermatophagoides-sIgE levels, and Aspergillus-sIgE levels were plotted against the titer of serum tIgE. P and r two statistical values for the linear regression are indicated.
